# Physical and technical demands of offence, defence, and contested phases of play in Australian Football

**DOI:** 10.1186/s13102-022-00425-1

**Published:** 2022-03-01

**Authors:** Christopher Wing, Nicolas H. Hart, Fadi Ma’ayah, Kazunori Nosaka

**Affiliations:** 1 Centre for Human Performance, School of Medical and Health Sciences, Edith Cowan University, 270 Joondalup Drive, Joondalup, WA 6027 Australia; 2 Exercise Medicine Research Institute, Edith Cowan University, Joondalup, WA Australia; 3 Institute for Health Research, University of Notre Dame Australia, Fremantle, WA Australia; 4 College of Nursing and Health Science, Flinders University, Adelaide, SA Australia; 5 School of Education, Curtin University, Bentley, WA Australia

**Keywords:** Match analysis, Microsensor technology, Physical performance, Performance analysis

## Abstract

**Background:**

This study compared the physical demands and effect of field location for different phases of play (offence, defence and contested), and examined the physical and technical demands of successful and unsuccessful phases of play during Australian Football matches.

**Methods:**

Global positioning system (GPS) and technical performance data were collected from 32 male Australian Football players in one club over 19 games in the 2019 season. The GPS data was aligned with phases of play acquired using Champion Data. Linear mixed models were used to detect differences between phases of play and field location which were further contextualized using Cohen’s *d* effect size.

**Results:**

Physical demands were greatest (*p* < 0.001) in defensive phases for backs (ES 0.61 to 1.42), and offensive phases for midfielders (ES 0.65 to 0.96) and forwards (ES 0.84 to 1.94). Additionally, distance and high-speed running were lowest in contested phases irrespective of playing position. Distance and high-speed running were greatest in larger field locations (e.g., full ground). No pattern was evident for accelerations or decelerations. Successful offensive plays demonstrated greater physical and technical outputs for midfielders and forwards, whereas the opposite was found for backs. Physical output was largely greater in unsuccessful defensive plays for all positions; however, the rate of tackles and marks was greater during successful defence.

**Conclusion:**

These findings enable a greater understanding of the demands of Australian Football matches, and can be utilized to inform both representative training design, and the evaluation of player performance.

## Background

Australian Football (AF) is an intermittent type of sport between two teams of 18 players, plus four upon the interchange bench, where the aim is to transfer the ball through kicks and handballs to create a scoring opportunity, with six points awarded for a goal, and one point awarded for a behind. During official matches, AF players frequently travel further than 12 km, with around 2 km of this recorded at high speed (> 14.4 km·h^−1^) [1]. The development of wearable microsensor technology devices, consisting of inertial measurement units (containing accelerometers, gyroscopes, and magnetometers) and global-positioning systems (GPS), has enabled the accurate and valid assessment of these running demands [2–5].

Typically, running demands are reported across a whole game, halves, and individual quarters [1, 6, 7]. However, AF is characterised by periods of offence, defence, and contest/dispute (i.e., periods where neither team has secured possession of the ball), which may have differing physical demands [8–12]. Gronow et al. [8] assessed these demands, demonstrating that backs spent a greater percentage of time performing high-intensity running without the ball, forwards with the ball, whilst midfielders demonstrated a more evenly distributed effort. More recently, Rennie et al. [11] identified only trivial to small differences between offence and defence across several measures of running performance, but when distance was expressed relative to playing time, moderate to large increases were noted during offence and defence when compared to contested periods of play. However, it is not yet fully understood if these differences are better explained when the players are divided into their positional groups.

Oftentimes, coaches utilise training drills that aim to replicate particular phases of play (e.g., offence and defence), which are typically classified based upon the area size they are performed in (e.g., small-sided games, full ground drills) [13, 14]. However, little data exists in the literature to guide the intensity prescription of these specific training drills in AF. Vella et al. [9] demonstrated differences in distance and high-speed running during specific phases of play (e.g., offence) depending upon where the phase started (e.g., forward-50). However, this study only focused on where the phase began, and did not consider the end field location, which is problematic when translating this data to training [9]. Therefore, it appears prudent to assess the demands of the different phases of play based upon the field locations they are played within.

An assessment of successful versus unsuccessful periods of play (e.g., when a goal or behind is scored versus when a team losses possession) may prove useful for practitioners, coaches, and the players themselves. For example, in rugby union this form of analysis identified that forwards displayed greater relative high-speed running distances during successful compared to unsuccessful attacking 22 entries [15]. Additionally, it may be useful to assess if technical actions (e.g., tackles) are performed at a greater rate during successful or unsuccessful plays. However, analysis of this kind is currently lacking within the current AF literature.

The aims of this study were to: (1) compare the physical demands of different phases of play in AF, (2) assess the differences in these demands based upon field locations, and (3) compare the physical and technical demands of successful and unsuccessful plays. We hypothesised that differences would exist between phases of play, and that these would be dependent upon playing position. We also hypothesised that relative distance and high-speed demands would be greater in larger field locations (e.g., full ground), whereas accelerations and decelerations would be greater in smaller field locations (e.g., forward-50). Additionally, it was hypothesised that successful plays would be performed at a greater intensity (e.g., high metres per minute) than unsuccessful plays in offence and defence. Information of this kind will aid coaches to assess strategy and tactical performance, enhance feedback to players and inform the design and monitoring of representative practice.

## Methods

### Participants

GPS data was collected from 32 male sub-elite AF athletes (age 22.6 ± 2.9 years; mass 83.6 ± 7.8 kg; height: 184.0 ± 7.5 cm) from one club competing in the 2019 West Australian Football League (WAFL) season over 19 official games (15 regular season; 4 finals series). During the season, the team recorded 13 wins and 6 losses. Match samples (i.e., individual player match recordings) were removed if a player was injured and therefore unable to complete the match, or if there was a failure of the recording device, which included any recordings where there were any periods of clear loss of data capture. This resulted in a total of 370 match samples (average observations per player 11.6 ± 6.7; range of 1–19) included in the final analyses.

Players were divided into three positional groups based on where they spent the most on-field time in each individual match. This included backs (full and half line, n = 11; match samples = 123), forwards (full and half line, n = 18; match samples = 123), and midfielders (inside midfielders and wing position, n = 17; match samples = 124). These positional groups were chosen as they have a similar technical and tactical role during match’s (e.g., a backs primary role is to prevent the opposition from scoring) [16]. Due to the unique position of the ruckman, data pertaining to this position was removed from the final analyses. Participants were provided with study information and provided their written informed consent. The study was approved by the Human Research Ethics Committee of Edith Cowan University.

### Procedures

GPS data was collected using the Playertek device (Catapult Innovations, Melbourne, Australia) sampling at 10 Hz, which was turned on ~ 1 h before play to ensure adequate GPS lock, and worn within a specifically designed pouch, sewed into the playing shirt. The accuracy of these devices has been previously reported [17]. To reduce inter-unit variability, the players wore the same device throughout the season [4, 18]. The following GPS metrics were recorded: total running distance (m), high-speed running (HSR; > 18 km·h^−1^), accelerations (efforts > 3 m·s^−2^), and decelerations (efforts > 3 m·s^−2^). These thresholds were selected as they had been previously employed in AF populations [19–21]. The PlayerLoad™ metric was considered due to its reported ability to also capture non-running events, however, due to its strong correlation (*r* = 0.93) with total distance [22], it was considered that findings relating to PlayerLoad™ would mirror those of total distance, therefore adding no additional value to this study. All metrics were derived from the GPS component of the microsensor device, including accelerations and decelerations, which had a dwell time of 0.5 s. All metrics were expressed relative to playing time.

Following each match, devices were downloaded onto proprietary software (Playertek Cloud, Melbourne, Australia), with split times corresponding to specific phases of play manually entered across the GPS data. These were adapted from Alexander et al. [10], and included:

- *Offensive phases:* Initiated upon the study team securing possession of the ball until any of the following occurred: (1) the opposition gained possession of the ball, (2) a stoppage (ball-up or ball out of bounds), (3) a goal or behind were scored, or (4) a mark was taken or free awarded that directly led to a set shot at goal.
- *Defensive phases:* Initiated upon the opposition securing possession of the ball until any of the following occurred: (1) the study team gained possession of the ball, (2) a stoppage (ball-up or ball out of bounds), (3) a goal or behind were scored, or (4) a mark was taken or free awarded that directly led to a set shot at goal.
- *Contested phases:* A period from the beginning of an umpire re-start (ball-up, throw in, or centre bounce), where neither side is deemed to have secured possession of the ball, thus the ball remains in-dispute.

Instances where a set shot was missed, resulting in play on, a new phase of play was initiated from the time in which the set shot was taken, and was labelled in-line with the above criteria. These time periods were identified through a timeline of events provided by Champion Data (Melbourne, Australia): a company that supply statistics, such as events and associated time stamps, to the Australian Football League (AFL) and WAFL. Their data has previously produced acceptable levels of reliability and validity [23]. Additionally, previous research has demonstrated the coding of such events to show acceptable levels of accuracy [24].

The data were then exported to Microsoft Excel (Microsoft Cooperation, WA, USA, v. 2112), where it was first cleaned for analysis by removing all individual phases where a given player did not complete the full phase of play (e.g., was rotated on or off). For each match, all phases were summed so that each player had a match total for offensive, defensive, and contested phases. Offensive and defensive phases of play were then labelled as successful or unsuccessful based on the following criteria:

- *Offence:* Successful offensive phases were considered to be those where a goal or behind were scored by the study team, or a set shot was taken at goal by the study team. All other offensive phases were labelled as unsuccessful
- *Defence:* Successful defensive phases were identified as periods where the study team regained possession of the ball, or when a stoppage occurred (ball-up or out of bounds) to end an opposition attack. Where the opposition team scored a goal or behind, or took a set shot at goal, the defensive play was labelled as unsuccessful.

Additionally, each phase was given a field location based upon the area of the playing oval in which the ball was located. These locations were derived from the Champion Data coding, and were defined as:

- *Forward-50 (F50):* Only inside the attacking 50-m area.
- *Defensive-50 (D50):* Only inside the defensive 50-m area.
- *Midfield (MID):* Between the attacking and defensive 50-m area.
- *Attacking midfield (AMID):* Combination of both F50 and midfield.
- *Defensive midfield (DMID):* Combination of both D50 and midfield.
- *Full ground (FG):* When the ball travels from one 50-m area to the other.

Finally, offensive and defensive phases of play were contextualised with player technical actions, which were time matched from Champion Data time lines and reported per minute of playing time to allow for the differences in duration between phases, and included the following [16, 25];

- *Kick—*disposing of the ball with any part of the leg below the knee.
- *Handball—*disposing of the ball by a hand pass.
- *Tackle—*using physical contact to prevent a successful disposal.
- *Mark—*Catching a ball that has been kicked which has travelled > 15 m and has not been touched by another player.

### Statistical analyses

To assess for differences in physical output between different phases (offence, defence, and contested), linear mixed models were constructed (using the lmerTest package in R) where phases of play were entered as a fixed effect, while athlete and round identification (i.e., match number during the season) were included as random effects. A separate model was fitted for each construct of running performance and for each playing position. Additionally, the same linear mixed model structure was used to identify the differences of both successful and unsuccessful plays, where outcome was included as a fixed effect. To test for differences between locations (i.e., where all playing positions were pooled), an additional set of models were constructed where location and playing position were entered as fixed effects, and athlete and round identification as random effects. A separate model was fitted for each phase of play and construct of running performance. Where significant effects were observed, Tukey’s post-hoc test was used to make pairwise comparisons (using the emmeans package in R). The significance level was set at *p* < 0.05. Normality was confirmed through visual inspection of the residual plots. Differences were contextualised using Cohen’s *d* effect sizes (ES), and associated 95% confidence intervals, obtained using a customised spreadsheet, where ≤ 0.2, 0.21 to 0.6, 0.61 to 1.2, 1.21 to 2.0 and > 2.0 effect size magnitudes were classified as trivial, small, moderate, large and very large respectively [26]. All statistical analyses were performed using either Microsoft Excel or R software (R, v4.0.4, The R Foundation for Statistical Computing, Vienna, Austria).

## Results

### Phases of play

#### Backs

**Fig. 1:**
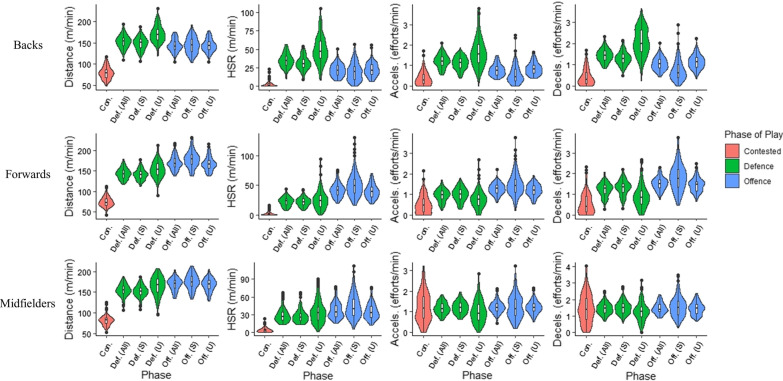
Physical output during specific phases of play. Key; HSR, high-speed running; Accels, accelerations; Decels, decelerations; Def. (all), all defensive phases; Def. (S), successful defensive phases; Def. (U), unsuccessful defensive phases; Off. (all), all offensive phases; Off. (S), successful offensive phases; Off. (U), unsuccessful offensive phases; Con, contested phases. The width of the violon plot indicates the approximate frequency of data points within the region

Defensive plays were greater than offensive (Fig. 1) for relative measures of distance (ES = 0.61 [− 0.24 to 1.47]), high-speed running (ES = 1.42 [0.49 to 2.36]), accelerations (ES = 1.38 [0.45 to 2.31]), and decelerations (ES = 1.33 [0.40 to 2.25]), all *p* < 0.001. Defensive and offensive plays were greater than contested plays (all *p* < 0.001) for relative measures of distance (ES = 5.03 [3.32 to 6.73] and 4.44 [2.89 to 6.00]), high-speed running (ES = 4.44 [2.88 to 5.99] and 2.85 [1.66 to 4.03]), accelerations (ES = 2.55 [1.43 to 3.68] and 1.35 [0.43 to 2.28]), and decelerations (ES = 2.94 [1.73 to 4.14], 1.81 [0.82 to 2.80]) respectively.

#### Forwards

Offensive plays were greater than defensive (Fig. 1) for relative measures of distance (ES = 1.76 [0.99 to 2.53]), high-speed running (ES = 1.94 [1.14 to 2.73]), accelerations (ES = 1.09 [0.39 to 1.79]), and decelerations (ES = 0.84 [0.16 to 1.53]), all *p* < 0.001. Offensive and defensive plays were greater than contested plays (all *p* < 0.001) for relative measures of distance (ES = 6.56 [4.91 to 8.22] and 5.45 [4.03 to 6.87]), high-speed running (ES = 4.54 [3.30 to 5.77] and 3.54 [2.49 to 4.59]), accelerations (ES = 2.08 [1.27 to 2.89] and 1.19 [0.48 to 1.90]), and decelerations (ES = 2.22 [1.39 to 3.05] and 1.53 [0.79 to 2.27]) respectively.

#### Midfielders

Offensive plays were greater than defensive (Fig. 1) for relative measures of distance (ES = 0.96 [0.25 to 1.67]), and high-speed running distance (ES = 0.65 [− 0.04 to 1.33]), all *p* < 0.001. Offensive and defensive plays were greater than contested plays (*p* < 0.001) for relative measures of distance (ES = 6.39 [4.73 to 8.04] and 5.41 [3.96 to 6.86]), and high-speed running distance (ES = 3.33 [2.29 to 4.37] and 2.92 [1.96 to 3.89]). There were no significant (p > 0.05) differences between any phases of play for accelerations and decelerations.

#### Successful versus unsuccessful offence

Physical output during successful and unsuccessful offence is illustrated in Fig. 1. Backs recorded no significant differences for relative distance (*p* = 0.745, ES = 0.03 [− 0.81 to 0.86]), however, relative measures of high-speed running (*p* = 0.001, ES = − 0.32 [− 1.16 to 0.52]), acceleration efforts (*p* < 0.001, ES = − 0.75 [− 1.61 to 0.12], and deceleration efforts (*p* < 0.001, ES = − 0.93 [− 1.81 to − 0.05]) were all greater in unsuccessful plays. Forwards recorded significantly greater (*p* < 0.001) relative measures of distance (ES = 0.56 [− 0.10 to 1.23]), high-speed running (ES = 0.66 [− 0.01 to 1.34]), acceleration efforts (ES = 0.61 [− 0.06 to 1.27]), and deceleration efforts (ES = 0.66 [− 0.01 to 1.34]) during successful plays. Midfielders performed significantly greater (*p* < 0.001) relative distance (ES = 0.44 [− 0.24 to 1.13]) and high-speed running (ES = 0.51 [− 0.17 to 1.20]) during successful plays, however no significant differences were noted for acceleration (*p* = 0.755, ES = 0.04 [− 0.63 to 0.71]) and deceleration efforts (*p* = 0.078, ES = 0.20 [− 0.48 to 0.87]) between the phases.

Technical actions per minute of playing time are reported in Table 1. Backs performed more kicks (ES = − 0.69 [− 1.55 to 0.17], handballs (ES = − 0.55 [− 1.40 to 0.30]), and marks (ES = − 0.40 [− 1.24 to 0.45]) during unsuccessful plays whereas forwards performed more kicks (ES = 0.79 [0.11 to 1.47]), handballs (ES = 0.33 [− 0.32 to 0.99]), and marks (ES = 1.09 [0.39 to 1.79]) in successful plays. Midfielders performed more kicks (ES = 0.51 [− 0.17 to 1.19]) and marks (ES = 0.48 [− 0.20 to 1.16]) in successful plays, but more handballs (ES = − 0.27 [− 0.94 to 0.41]) in unsuccessful plays.

**Table 1:** Technical actions per minute (Mean ± SD) and comparison statistics (ES and 95% CI) for successful and unsuccessful phases of play

Playing Position	Action	Offence			Defence		
		Successful	Unsuccessful	Comparison (E.S)	Successful	Unsuccessful	Comparison (E.S)
Backs	Handball/min	0.11 ± 0.15	0.18 ± 0.10	− 0.55 (− 1.40 to 0.30)	–	–	–
	Kick/min	0.22 ± 0.25	0.38 ± 0.21	− 0.69 (− 1.55 to 0.17)	–	–	–
	Mark/min	0.07 ± 0.11	0.11 ± 0.09	− 0.40 (− 1.24 to 0.45)	0.05 ± 0.06	0.00 ± 0.00	1.18 (0.27 to 2.08)
	Tackle/min	–	–	–	0.07 ± 0.05	0.04 ± 0.10	0.38 (− 0.46 to 1.22)
Forwards	Handball/min	0.25 ± 0.28	0.18 ± 0.10	0.33 (− 0.32 to 0.99)	–	–	–
	Kick/min	0.42 ± 0.31	0.23 ± 0.14	0.79 (0.11 to 1.47)	–	–	–
	Mark/min	0.29 ± 0.25	0.09 ± 0.07	1.09 (0.39 to 1.79)	0.01 ± 0.02	0.00 ± 0.00	0.71 (0.03 to 1.38)
	Tackle/min	–	–	–	0.11 ± 0.07	0.02 ± 0.08	1.20 (0.49 to 1.91)
Midfielders	Handball/min	0.28 ± 0.25	0.34 ± 0.20	− 0.27 (− 0.94 to 0.41)	–	–	–
	Kick/min	0.51 ± 0.35	0.37 ± 0.17	0.51 (− 0.17 to 1.19)	–	–	–
	Mark/min	0.17 ± 0.19	0.10 ± 0.08	0.48 (− 0.20 to 1.16)	0.01 ± 0.03	0.00 ± 0.00	0.47 (− 0.21 to 1.15)
	Tackle/min	–	–	–	0.14 ± 0.10	0.01 ± 0.05	1.64 (0.87 to 2.42)

#### Successful versus unsuccessful defence

Physical output during successful and unsuccessful defence is illustrated in Fig. 1. Backs recorded significantly greater (*p* < 0.001) relative measures of distance (ES = − 1.20 [− 2.11 to − 0.30]), high-speed running (ES = − 1.25 [− 2.16 to − 0.33]), acceleration (ES = − 0.94 [− 1.82 to − 0.06]), and deceleration efforts (ES = − 1.52 [− 2.47 to − 0.57]), during unsuccessful plays. Forwards performed greater relative measures of distance (*p* < 0.001, ES = − 0.75 [− 1.42 to − 0.07]) and high-speed running (*p* = 0.008, ES = − 0.29 [− 0.94 to 0.37] during unsuccessful plays, however, significantly (*p* < 0.001) greater measures of acceleration (ES = 0.61 [− 0.06 to 1.27]), and deceleration efforts (ES = 0.81 [0.13 to 1.48]) were noted in successful plays. Midfielders performed significantly (*p* < 0.001) greater relative distance (ES = − 0.67 [− 1.36 to 0.02]) and high-speed running (ES = − 0.51 [− 1.19 to 0.17]) in unsuccessful plays, but significantly (*p* < 0.001) greater acceleration (ES = 0.45 [− 0.23 to 1.13]), and deceleration (ES = 0.50 [− 0.18 to 1.18]) efforts in successful plays. All positions performed more technical actions during successful defensive plays (Table 1), backs (marks (ES = 1.18 [0.27 to 2.08]) and tackles (ES = 0.38 [− 0.46 to 1.22])), forwards (marks (ES = 0.71 [0.03 to 1.38]) and tackles (ES = 1.20 [0.49 to 1.91])), and midfielders (marks (ES = 0.47 [− 0.21 to 1.15]) and tackles (ES = 1.64 [0.87 to 2.42])).

### Field location

#### Offence

**Fig. 2:**
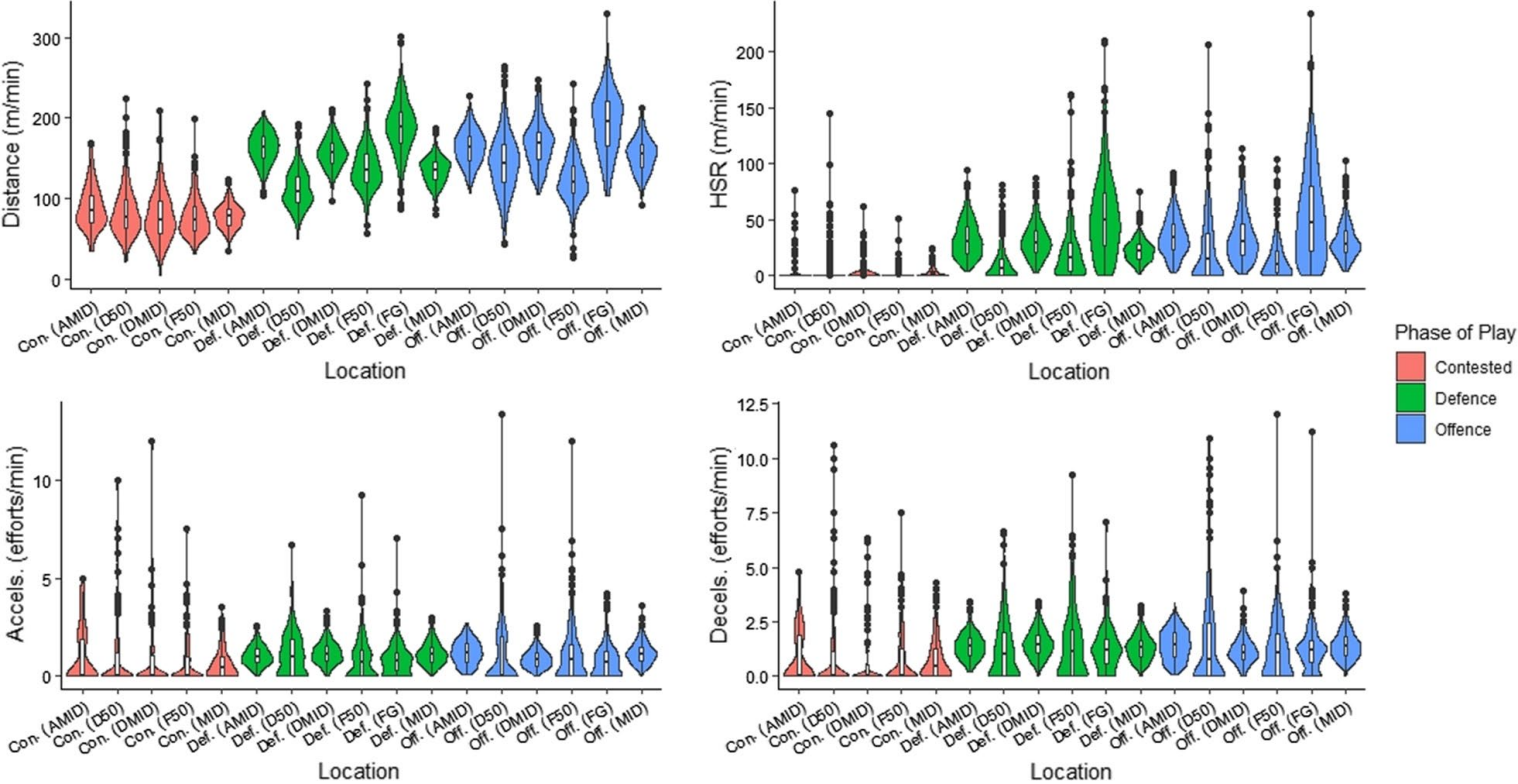
Physical output by field location. Key; HSR, high-speed running; Accels, accelerations; Decels, decelerations; Def, defence; Off, offence; Con, contested; F50, forward-50; AMID, attacking midfield; MID, midfield; DMID, defensive midfield; D50, defensive-50; FG, full-ground. The width of the violon plot indicates the approximate frequency of data points within the region

Main findings (Fig. 2 and Table 2) demonstrated that measures of relative distance and high-speed running were reflective of field location, where the larger the location, the greater the metres per minute recorded. Specifically, the greatest values were noted for full-ground, which were significantly (*p* < 0.001) greater than all other locations, whilst the lowest values were recorded for forward-50, which were significantly (*p* < 0.001) lower than all other locations. Conversely, acceleration efforts per minute were lowest in the largest field location (full-ground), which were significantly (*p* < 0.001) lower than all other locations except for defensive midfield. Deceleration efforts per minute were greatest in the defensive-50 location, which were significantly (*p* < 0.05) greater than forward-50, defensive midfield and full ground locations. Relative deceleration efforts were lowest in the defensive midfield location, which were significantly (*p* < 0.05) lower than attacking midfield, defensive-50 and midfield locations.

**Table 2:** Comparison statistics (p values and effect sizes (E.S and 95% CI) between field locations for phases of play

Location comparison	Comparison statistic	Offence				Defence			
		Dist/min	HSR/min	Accels/min	Decels/min	Dist/min	HSR/min	Accels/min	Decels/min
F50 v AMID	*P* value E.S 95% CI	< 0.001 − 1.56 (− 2.12 to − 1.00)	< 0.001 − 1.19 (− 1.73 to − 0.66)	1.000 − 0.02 (− 0.51 to 0.47)	0.280 − 0.16 (− 0.65 to 0.33)	< 0.001 − 1.06 (− 1.59 to − 0.54)	< 0.001 − 0.59 (− 1.09 to − 0.09)	0.078 − 0.19 (− 0.68 to 0.30)	0.859 0.07 (− 0.42 to 0.56)
F50 v D50	*P* value E.S 95% CI	< 0.001 − 0.60 (− 1.10 to − 0.10)	< 0.001 − 0.38 (− 0.87 to 0.11)	0.907 0.05 (− 0.44 to 0.54)	0.048 − 0.14 (− 0.63 to 0.35)	< 0.001 1.02 (0.50 to 1.54)	< 0.001 0.53 (0.03 to 1.03)	< 0.001 − 0.26 (− 0.76 to 0.23)	0.017 0.17 (− 0.32 to 0.66)
F50 v DMID	*P* value E.S 95% CI	< 0.001 − 1.51 (− 2.06 to − 0.95)	< 0.001 − 1.02 (− 1.54 to − 0.50)	< 0.001 0.27 (− 0.22 to 0.77)	0.866 0.09 (− 0.40 to 0.58)	< 0.001 − 0.80 (− 1.31 to − 0.29)	< 0.001 − 0.55 (− 1.05 to − 0.05)	< 0.001 − 0.38 (− 0.87 to 0.11)	0.998 − 0.03 (− 0.52 to 0.46)
F50 v FG	*P* value E.S 95% CI	< 0.001 − 2.04 (− 2.65 to − 1.44)	< 0.001 − 1.27 (− 1.80 to − 0.73)	< 0.001 0.32 (− 0.18 to 0.81)	1.000 0.01 (− 0.48 to 0.50)	< 0.001 − 1.63 (− 2.20 to − 1.07)	< 0.001 − 1.04 (− 1.57 to − 0.52)	0.997 0.03 (− 0.46 to 0.52)	0.038 0.17 (− 0.32 to 0.66)
F50 v MID	*P* value E.S 95% CI	< 0.001 − 1.20 (− 1.73 to − 0.67)	< 0.001 − 1.00 (− 1.52 to − 0.48)	1.000 0.00 (− 0.49 to 0.49)	0.329 − 0.16 (− 0.65 to 0.33)	0.132 0.16 (− 0.33 to 0.65)	0.931 − 0.07 (− 0.56 to 0.42)	< 0.001 − 0.32 (− 0.82 to 0.17)	0.399 0.12 (− 0.37 to 0.61)
AMID v D50	*P* value E.S 95% CI	< 0.001 0.65 (0.14 to 1.15)	< 0.001 0.48 (− 0.02 to 0.97)	0.774 0.08 (− 0.41 to 0.57)	0.975 − 0.04 (− 0.53 to 0.45)	< 0.001 2.36 (1.72 to 3.00)	< 0.001 1.47 (0.92 to 2.03)	0.175 − 0.15 (− 0.64 to 0.34)	0.306 0.15 (− 0.34 to 0.65)
AMID v DMID	*P* value E.S 95% CI	0.457 − 0.13 (− 0.62 to 0.36)	0.967 0.07 (− 0.42 to 0.56)	< 0.001 0.62 (0.11 to 1.12)	0.014 0.44 (− 0.06 to 0.94)	< 0.001 0.36 (− 0.13 to 0.86)	0.983 0.06 (− 0.43 to 0.55)	0.034 − 0.31 (− 0.80 to 0.19)	0.599 − 0.19 (− 0.68 to 0.30)
AMID v FG	*P* value E.S 95% CI	< 0.001 − 0.95 (− 1.47 to − 0.44)	< 0.001 − 0.58 (− 1.08 to − 0.08)	< 0.001 0.57 (0.07 to 1.07)	0.202 0.22 (− 0.27 to 0.71)	< 0.001 − 0.91 (− 1.42 to − 0.39)	< 0.001 − 0.71 (− 1.22 to − 0.21)	0.022 0.27 (− 0.22 to 0.77)	0.456 0.18 (− 0.32 to 0.67)
AMID v MID	*P* value E.S 95% CI	< 0.001 0.43 (− 0.06 to 0.93)	0.043 0.29 (− 0.21 to 0.78)	1.000 0.04 (− 0.45 to 0.53)	1.000 0.01 (− 0.48 to 0.50)	< 0.001 1.60 (1.04 to 2.17)	< 0.001 0.79 (0.28 to 1.30)	0.304 − 0.22 (− 0.71 to 0.27)	0.975 0.09 (− 0.40 to 0.58)
D50 v DMID	*P* value E.S 95% CI	< 0.001 − 0.70 (− 1.20 to − 0.19)	< 0.001 − 0.40 (− 0.90 to 0.09)	0.023 0.20 (− 0.29 to 0.69)	< 0.001 0.23 (− 0.26 to 0.72)	< 0.001 − 2.10 (− 2.71 to − 1.49)	< 0.001 − 1.44 (− 1.99 to − 0.89)	0.991 − 0.04 (− 0.53 to 0.45)	0.004 − 0.26 (− 0.76 to 0.23)
D50 v FG	*P* value E.S 95% CI	< 0.001 − 1.30 (− 1.84 to − 0.76)	< 0.001 − 0.85 (− 1.36 to − 0.33)	< 0.001 0.24 (− 0.25 to 0.73)	0.030 0.16 (− 0.33 to 0.65)	< 0.001 − 2.61 (− 3.28 to − 1.95)	< 0.001 − 1.52 (− 2.08 to − 0.96)	< 0.001 0.32 (− 0.17 to 0.82)	1.000 − 0.02 (− 0.51 to 0.47)
D50 v MID	*P* value E.S 95% CI	< 0.001 − 0.34 (− 0.84 to 0.15)	< 0.001 − 0.29 (− 0.79 to 0.20)	0.918 − 0.06 (− 0.55 to 0.43)	0.959 0.05 (− 0.04 to 0.54)	< 0.001 − 1.09 (− 1.61 to − 0.56)	< 0.001 − 0.96 (− 1.48 to − 0.44)	1.000 0.02 (− 0.47 to 0.51)	0.771 − 0.10 (− 0.59 to 0.39)
DMID v FG	*P* value E.S 95% CI	< 0.001 − 0.78 (− 1.29 to − 0.27)	< 0.001 − 0.61 (− 1.11 to − 0.10)	0.928 0.11 (− 0.38 to 0.60)	0.929 − 0.10 (− 0.59 to 0.39)	< 0.001 − 1.17 (− 1.70 to − 0.64)	< 0.001 − 0.75 (− 1.26 to − 0.24)	< 0.001 0.50 (0.01 to 1.00)	0.009 0.31 (− 0.18 to 0.81)
DMID v MID	*P* value E.S 95% CI	< 0.001 0.51 (0.01 to 1.00)	0.286 0.19 (− 0.30 to 0.68)	< 0.001 − 0.60 (− 1.10 to − 0.10)	0.019 − 0.45 (− 0.94 to 0.05)	< 0.001 1.27 (0.74 to 1.81)	< 0.001 0.74 (0.23 to 1.24)	0.942 0.09 (− 0.40 to 0.58)	0.174 0.27 (− 0.22 to 0.76)
FG v MID	*P* value E.S 95% CI	< 0.001 1.26 (0.72 to 1.79)	< 0.001 0.75 (0.25 to 1.26)	< 0.001 − 0.55 (− 1.05 to − 0.05)	0.243 − 0.22 (− 0.71 to 0.28)	< 0.001 2.03 (1.43 to 2.63)	< 0.001 1.14 (0.61 to 1.67)	< 0.001 − 0.44 (− 0.93 to 0.06)	0.890 − 0.10 (− 0.59 to 0.39)

Location comparison	Comparison statistic	Contested			
		Dist/min	HSR/min	Accels/min	Decels/min
F50 v AMID	*P* value E.S 95% CI	< 0.001 − 0.46 (− 0.96 to 0.03)	< 0.001 − 0.47 (− 0.97 to 0.02)	0.341 − 0.22 (− 0.71 to 0.27)	0.936 − 0.08 (− 0.57 to 0.41)
F50 v D50	*P* value E.S 95% CI	0.014 − 0.26 (− 0.75 to 0.23)	0.031 − 0.23 (− 0.72 to 0.26)	0.371 − 0.15 (− 0.64 to 0.34)	0.999 − 0.03 (− 0.52 to 0.46)
F50 v DMID	*P* value E.S 95% CI	0.997 − 0.02 (− 0.51 to 0.47)	0.617 − 0.19 (− 0.68 to 0.31)	0.990 − 0.05 (− 0.54 to 0.44)	0.852 0.08 (− 0.41 to 0.57)
F50 v FG	*P* value E.S 95% CI	–	–	–	–
F50 v MID	*P* value E.S 95% CI	0.921 − 0.02 (− 0.51 to 0.47	0.702 − 0.28 (− 0.77 to 0.22)	0.973 0.00 (− 0.49 to 0.49)	0.999 − 0.04 (− 0.53 to 0.45)
AMID v D50	*P* value E.S 95% CI	0.006 0.15 (− 0.34 to 0.64)	0.015 0.21 (− 0.29 to 0.70)	0.934 0.03 (− 0.46 to 0.53)	0.966 0.04 (− 0.45 to 0.53)
AMID v DMID	*P* value E.S 95% CI	< 0.001 0.39 (− 0.10 to 0.88)	0.006 0.32 (− 0.17 to 0.81)	0.650 0.14 (− 0.35 to 0.63)	0.615 0.16 (− 0.33 to 0.65)
AMID v FG	*P* value E.S 95% CI	–	–	–	–
AMID v MID	*P* value E.S 95% CI	< 0.001 0.53 (0.03 to 1.02)	< 0.001 0.37 (− 0.12 to 0.87)	0.153 0.25 (− 0.24 to 0.74)	0.964 0.05 (− 0.44 to 0.54)
D50 v DMID	*P* value E.S 95% CI	0.193 0.21 (− 0.28 to 0.71)	0.902 0.09 (− 0.40 to 0.58)	0.869 0.09 (− 0.40 to 0.58)	0.744 0.10 (− 0.39 to 0.59)
D50 v FG	*P* value E.S 95% CI	–	–	–	–
D50 v MID	*P* value E.S 95% CI	< 0.001 0.28 (− 0.22 to 0.77)	0.416 0.11 (− 0.38 to 0.60)	0.082 0.17 (− 0.32 to 0.66)	1.000 0.00 (− 0.49 to 0.49)
DMID v FG	*P* value E.S 95% CI	–	–	–	–
DMID v MID	*P* value E.S 95% CI	0.846 0.00 (− 0.49 to 0.49)	0.993 0.02 (− 0.47 to 0.51)	0.869 0.06 (− 0.43 to 0.55)	0.742 − 0.13 (− 0.62 to 0.36)
FG v MID	*P* value E.S 95% CI	–	–	–	–

Key, Dist: distance; HSR, high-speed running; Accels, accelerations; Decels, decelerations; F50, forward-50; AMID, attacking midfield; DMID, defensive midfield; MID, midfield; D50, defensive-50; FG, full ground

#### Defence

Main findings (Fig. 2 and Table 2) demonstrated that relative distance and high-speed running was again greatest in the full-ground location, which was significantly (*p* < 0.001) higher than all other locations. The lowest relative values of distance and high-speed running were noted in the defensive-50, where they were significantly (*p* < 0.001) lower than all other locations. Relative acceleration efforts were lowest in the full-ground location, which were significantly (*p* < 0.05) less than all locations except for forward-50. Few significant differences were noted for relative deceleration efforts, where only those recorded in defensive midfield and forward-50 were significantly (*p* < 0.05) greater than full-ground and defensive-50 locations.

#### Contested

Only few significant differences were noted between field locations during contested phases of play (Fig. 2 and Table 2). Relative measures of distance and high-speed running were all significantly (*p* < 0.05) greater in attacking midfield compared to all other comparison locations. Further, relative distance and high-speed running were significantly (*p* < 0.05) greater in defensive-50 versus forward-50, and for distance in defensive-50 versus midfield. There were no significant differences in acceleration and deceleration efforts.

## Discussion

Our hypotheses were that there would be significant differences between the phases of play and that these differences would be dependent upon playing position. The results demonstrated that the physical demands were greater in defence for the backs, and in offence for the forwards, whereas midfielders performed greater distance and high-speed running in offence, without significant differences in accelerations and decelerations between the phases. In-line with our second hypothesis, measures of distance and high-speed running were greater in the larger field locations, however, no specific pattern was noted for accelerations and decelerations. Our final hypothesis was that successful plays would be performed at a greater intensity than unsuccessful plays. This was the case during successful offence for both midfielders (distance and high-speed distance) and forwards (all metrics). However, measures of distance and high-speed distance were greater during unsuccessful defence for all positional groups.

Comparisons between phases of play highlighted the prevalence of significant differences that varied depending upon playing position. Backs displayed higher outputs in defensive phases, whilst forwards displayed higher outputs in offensive phases for all measured metrics, which is indicative of their positional role. However, despite midfielders performing significantly more relative distance and high-speed running in offence, they recorded no significant differences in acceleration and deceleration efforts. Additionally, with the exception of distance amongst the backs, midfielders recorded smaller effect size comparisons than the other two playing positions, which may indicate their physical output is more evenly distributed between offence and defence, in line with previous findings and accordance with their positional role [8]. Furthermore, the effect sizes reported between offence and defence in this study (moderate to large) are greater than those reported in the study by Rennie et al. [11] (trivial to small), potentially owing to the delineation of physical output into positional groups within this study, or the differences in coding that exist within the literature regarding contested plays [8, 10, 12, 24].

Despite the differences in coding, contested plays were performed at a lower metres per minute than offence and defence, in line with findings from previous research [24]. This finding is unsurprising and owed to contested plays being located at stoppages, where players are required to jostle or wrestle for possession of the ball, thus reducing the requirement to perform locomotion. This is highlighted in previous research using spatiotemporal data, where the density of players was greatest during contested phases of play [12]. An interesting finding within this study relates to acceleration and deceleration efforts relative to playing time amongst the midfielders during contested phases of play, where there were no significant differences observed in comparison to offence and defence. This is likely attributed to the role of midfielders at stoppages who are centred around the ball, thus increasing their requirement to accelerate and decelerate, which is not the case for backs and forwards [16]. This is supported by Rennie et al. [11] who reported that acceleration and deceleration load is higher during contested play, with the findings of our study indicating that this demand is greatest upon the midfield playing group. It is important for coaches to be cognizant of these differences that exist between the three phases of play, as training is often prescribed with the intention of practicing specific elements of a game (e.g., stoppages), and also within position groups (e.g., line training) [27]. Therefore, having a greater understanding of the physical demands of each phase of play, per playing position, can ensure the appropriate training intensity is matched to specific training design.

When studying successful versus unsuccessful offensive phases, midfielders (distance and high-speed running) and forwards (all metrics) were the only groups to recorded significantly greater outputs during successful offensive plays, whilst backs generally recorded greater values during unsuccessful play. The findings for midfielders and forwards are in line with those reported within rugby union, where relative high-speed running was greater during successful attacking 22 entries [15]. This finding may potentially indicate that successful play relies upon fast ball movement, where players are also required to move at speed to either spread (to create an opportunity for an effective disposal) or to carry the ball [28]. This is somewhat supported by Lane et al. [29] who suggest that slow ball movement leads to greater congestion and lower scoring. This may have important implications for representative training, where drills aimed to improve a team’s offensive play, such as ball movement drills, should replicate the intensities derived from successful match performance in order to promote positive transfer to competition [27, 28, 30]. Additionally, increasing a player’s physical capacity in order to match these demands may also prove beneficial, this is particularly pertinent amongst the midfield and forward positional groups.

Similar findings were evident for technical actions during offensive phases, where backs performed kicks, handballs, and marks at a greater rate during unsuccessful plays, indicating that their impact on successful offensive performance is somewhat limited. Conversely, midfielders (kicks and marks) and forwards (kicks, handballs, and marks) performed technical actions at a greater rate during successful plays, highlighting the need to combine skill execution and enhanced running performance to achieve superior offensive outcomes. This combined approach to training appears to be of high importance, particularly when previous research has shown that kicking accuracy is reduced when kicking to a marked target as well as when the kicker has reduced time in possession, and is under increased opposition pressure [31]. However, it has also been reported that AF players are underexposed to these constraints during representative practice [30]. Therefore, there is a need to create training environments where skills (e.g., kicks) are performed under match conditions, which is likely to improve perception action coupling as well as player decision making, and lead to greater transfer to competitive matches [30, 32]. Interestingly, midfielders performed handballs at a higher rate during unsuccessful plays, potentially indicating greater importance of kicking to successful outcomes. The importance of kicking performance to successful match outcomes has been previously demonstrated, where it has been reported that team kick (and goal conversion) values were the two biggest contributors to successful match outcome [33]. This may indicate that handballing offers a greater chance for the opposition to regain possession of the ball or force a stoppage, as a player can be tackled upon receiving a handball. This is not the case following a kick that is secured via a mark, where the receiving player is afforded a short period of time to perform a secondary kick, either to transfer the ball to a teammate or to take a shot at goal, which is unimpeded by opposition players. Previous research lends some support to this theory, where the frequency of handballs performed under physical pressure (3.1 ± 1.7) was greater than that of kicks (1.19 ± 0.83) [30]. This information could be used to benchmark player performance, where a desired kick: handball ratio (number of kicks relative to number of handballs) may be targeted by coaching staff. The value of the kick-to-handball ratio has been highlighted by Robertson et al. [33], where winning teams demonstrated a higher kick-to-handball ratio compared to losing teams. However, it should be noted that this finding may be specific to the style of play of the study team, and may be relevant to the effectiveness of disposals (i.e. accurately reaching the intended target) and therefore not generalisable to the wider AF population where teams may have differing styles of play, as demonstrated by previous research [34].

Comparisons of successful and unsuccessful defensive phases highlighted that measures of distance and high-speed running were greater for all positions in unsuccessful phases. It is possible that during unsuccessful defensive play, opposition ball movement may be quicker, increasing the need for the defensive team to chase the ball and opposition [29]. This may be particularly evident during turn-over, where the team now defending is likely to be caught out of position. These occurrences are potentially heightened during unsuccessful play, as score from turnover has been previously identified as a contributing factor to match outcome [35]. Furthermore, Vella et al. [9] noted that relative high-speed running distances were greatest when defensive phases began with an intercept, adding further evidence to this theory. Conversely, acceleration and deceleration efforts were greater during successful defensive plays for midfielders and forwards. Although this may indicate the importance of accelerating and decelerating to perform successfully in defence for these positional groups, it should be noted that it is difficult to ascertain if these measures are an indication of an athlete changing direction or performing a tackle and collision [11]. This is particularly relevant when tackles were performed at a greater rate during successful defensive plays within these positional groups. Additionally, marks and tackles were performed at a greater rate during successful plays for all positional groups, highlighting that the completion of these actions likely contributes more to successful play than physical output. Furthermore, the completion of tackles appears to be especially important for midfielders, where the effect size was calculated to be large. Therefore, it appears prudent that coaches afford dedicated training time to tackling and marking in defensive scenarios. As previously mentioned, these should be performed under match conditions (e.g., intensity), in order to facilitate positive transfer to performance.

Comparisons between field location demonstrated that relative measures of distance and high-speed running were greatest for both offence and defence in the largest field locations (full ground and attacking and defensive midfield), and lowest in the smallest field locations (defensive and forward-50), with exception of relative distance during defence where measures were greater in the forward-50 than midfield. This larger area potentially affords athletes with less congestion (i.e., number of players in proximity), as well as a larger distance to accelerate to higher velocity running, allowing them to produce superior relative running performance and greater velocities [16, 36]. Therefore, coaches should be cognizant to these outputs when devising and monitoring training drills in order to adequately meet these physical demands, where drills played on a full ground with reduced numbers are likely to elicit higher relative distances and high-speed running to those played in smaller areas. This is supported by previous research which demonstrated that the implementation of small sided games on larger pitch areas leads to greater distance and high-speed running performed by AF players [36].

Although it is expected that acceleration and deceleration efforts would increase in smaller field locations, this was not always the case, where there appeared to be no specific pattern demonstrated. There was evidence of both larger (e.g., attacking, and defensive midfield) and smaller (e.g., defensive and forward-50) pitch locations showing both comparably higher and lower measures for these metrics. However, previous research in soccer has demonstrated that small-sided games played on medium and larger pitches showed greater acceleration demand than those played on small pitches, although it should be noted that the medium sized pitch demonstrated the highest demand [37]. Another study in soccer populations also support this, where the number of high accelerations and decelerations was similar (*p* > 0.05) between drill sizes [38]. Combined, this evidence may suggest that if coaches wish to expose athletes in training to similar acceleration and deceleration efforts to those experienced during a game, area size may not be of primary concern.

Finally, there were few differences in physical output during contested phases of play, which is unsurprising considering these phases represent a time where the ball is somewhat locked into a contest during a stoppage. These were greatest in the attacking-midfield location; however, this finding is somewhat difficult to explain and could be potentially owed to the effort of the attacking team attempting to force the ball into the forward-50 location, and therefore closer to goal. Additionally, it should be noted that the effect size comparisons were only trivial to small.

## Limitations and future directions

This study had some limitations which should be addressed. Firstly, the players were grouped into three positional groups (backs, midfield and forwards). Despite the use of general positional groups being standard practice within AF research [16, 39, 40], the running demands of AF players can be delineated further into smaller groups (e.g., half backs and full backs), which may provide a greater level of detail [1]. This would require a substantially larger sample size, which if achieved, may also enable the inclusion of the ruck position. We have included four primary GPS metrics; however, as plethora of metrics is available to practitioners, it may be useful to include some of these in any future studies (e.g., collisions, sprint efforts.). As with all single study designs, the applicability of these findings to other AF teams may be limited. This is due to the differing styles of play that exist across AF teams (e.g., some may play an open, fast paced style, while others may adopt a more contested approach) [34]. It may be helpful for future studies to include data from multiple teams to assess the impact of playing style on the physical and technical characteristics of the three phases of play, and in-particular if style of play has an effect on the characteristics of successful play. It may also be interesting to include data from both teams competing in the same match, which may be provide greater insights into the determinants of successful play. For example, it is interesting to know the differences in physical output between the offensive and defensive team during periods of successful offence.

## Practical applications

The findings of this study have several practical applications to those practitioners working with AF players. The findings of the present study indicate that there are differences in the physical and technical demands dependent on the phase of play and playing position. This supports the need for specificity of representative training design in order to prepare players for both the specific phases of play and the likely role they will play in that phase, based on their playing positions. Additionally, it may be beneficial to subject players to the requirements of multiple positional groups, so that they are adequately prepared should they be required to play multiple positions. As field location plays a significant role in a players physical output, field dimensions may be manipulated during AF training sessions to match these specific outputs. In both cases, training drills and sessions can be appropriately monitored to ensure adequate training intensity by using the data derived from this study. It was highlighted that increased running intensities were noted amongst midfielders and forwards during successful offensive plays. Therefore, it appears prudent to develop AF players physical capacity to a level in which they can meet these demands to potentially increase the likelihood of successful offensive outcomes. Finally, as successful defence appears to be reliant upon the completion of technical actions (e.g., tackles and marks), and therefore potentially tactical understanding, coaches should focus upon developing these areas during representative practice that is aimed at enhancing defensive outcomes.

## Conclusion

Our findings can provide coaches and practitioners with a greater understanding of the physical demands of AF match play based on player position, once delineated into phases of play and field locations. Specifically, the physical demands of match play are greater in defence for backs, and in offence for midfielders and forwards, whilst contested phases produced the lowest physical demands. Additionally, measures of distance and high-speed running are greater in both offence and defence when phases are performed in larger field locations. Additionally, successful offensive phases appear to be dependent on both physical output and the performance of technical skills amongst midfielders and forwards, whereas successful defensive play appears to rely more heavily on the performance of marks and tackles. This information could be used to benchmark player performance and to guide the design and monitoring of representative practice.

## Data Availability

Due to the agreement with the football club, the raw data set associated with this manuscript is not available publicly, so supporting data is not available.

## Abbreviations

AF: Australian Football
WAFL: West Australian Football League
F50: Forward-50
D50: Defensive-50
MID: Midfield
AMID: Attacking midfield
DMID: Defensive midfield
FG: Full ground
ES: Effect size
Dist: Distance
HSR: High-speed running
Accels: Accelerations
Decels: Decelerations
Def: Defensive
Off: Offensive
Con: Contested

## References

[1] Coutts AJ, Kempton T, Sullivan C, Bilsborough J, Cordy J, Rampinini E (2015). Metabolic power and energetic costs of professional Australian football match-play. J Sci Med Sport.

[2] Scott M, Scott T, Kelly V (2016). The validity and reliability of global positioning systems in team sport: a brief review. J Strength Cond Res..

[3] Johnston RJ, Watsford ML, Kelly SJ, Pine MJ, Spurrs RW (2014). Validity and interunit reliability of 10 Hz and 15 Hz GPS units for assessing athlete movement demands. J Strength Cond Res.

[4] Thornton HR, Nelson AR, Delaney JA, Serpiello FR, Duthie GM (2019). Interunit reliability and effect of data-processing methods of global positioning systems. Int J Sports Physiol Perform.

[5] Varley MC, Fairweather IH, Aughey RJ (2012). Validity and reliability of GPS for measuring instantaneous velocity during acceleration, deceleration, and constant motion. J Sports Sci.

[6] Johnston RJ, Watsford ML, Pine MJ, Spurrs RW, Murphy A, Pruyn EC (2012). Movement demands and match performance in professional Australian football. Int J Sports Med.

[7] Coutts AJ, Quinn J, Hocking J, Castagna C, Rampinini E (2010). Match running performance in elite Australian Rules Football. J Sci Med Sport.

[8] Gronow D, Dawson B, Heasman J, Rogalski B, Peeling P (2014). Team movement patterns with and without ball possession in Australian Football League players. Int J Perform Anal Sport.

[9] Vella A, Clarke AC, Kempton T, Ryan S, Holden J, Coutts AJ (2021). Possession chain factors influence movement demands in elite Australian football match-play. Sci Med Footb.

[10] Alexander JP, Spencer B, Mara JK, Robertson S (2019). Collective team behaviour of Australian Rules football during phases of match play. J Sports Sci.

[11] Rennie MJ, Kelly SJ, Bush S, Spurrs RW, Austin DJ, Watsford ML (2020). Phases of match-play in professional Australian Football: distribution of physical and technical performance. J Sports Sci.

[12] Alexander JP, Spencer B, Sweeting AJ, Mara JK, Robertson S (2019). The influence of match phase and field position on collective team behaviour in Australian Rules football. J Sports Sci.

[13] Boyd LJ, Ball K, Aughey RJ (2013). Quantifying external load in australian football matches and training using accelerometers. Int J Sports Physiol Perform.

[14] Loader J, Montgomery PG, Williams MD, Lorenzen C, Kemp JG (2012). Classifying training drills based on movement demands in Australian Football. Int J Sports Sci Coach.

[15] Tierney P, Tobin DP, Blake C, Delahunt E (2017). Attacking 22 entries in rugby union: running demands and differences between successful and unsuccessful entries. Scand J Med Sci Sports.

[16] Johnston RD, Black GM, Harrison PW, Murray NB, Austin DJ (2018). Applied sport science of Australian football: a systematic review. Sport Med.

[17] Mooney T, Malone S, Izri E, Dowling S, Darragh IAJ (2021). The running performance of elite U20 Gaelic football match-play. Sport Sci Health.

[18] Beato M, Devereux G, Stiff A (2018). Validity and reliability of global positioning system units (STATSports Viper) for measuring distance and peak speed in sports. J Strength Cond Res.

[19] Edwards T, Piggott B, Joyce C, Chivers P (2015). The relationship between two measures of physical capacity and match performance in semi-professional Australian rules football. J Aust Strength Cond.

[20] Gastin PB, Hunkin SL, Fahrner B, Robertson S (2019). Deceleration, Acceleration, and Impacts are strong contributors to muscle damage in professional Australian Football. J Strength Cond Res.

[21] Janetzki SJ, Bourdon PC, Norton KI, Lane JC, Bellenger CR (2021). Evolution of physical demands of Australian Football League matches from 2005 to 2017: a systematic review and meta-regression. Sport Med - Open..

[22] Scott BR, Lockie RG, Knight TJ, Clark AC, De Jonge XAKJ (2013). A comparison of methods to quantify the in-season training load of professional soccer players. Int J Sports Physiol Perform.

[23] Robertson S, Gupta R, McIntosh S (2016). A method to assess the influence of individual player performance distribution on match outcome in team sports. J Sports Sci.

[24] Rennie MJ, Watsford ML, Spurrs RW, Kelly SJ, Pine MJ (2018). Phases of match-play in professional Australian Football: Descriptive analysis and reliability assessment. J Sci Med Sport.

[25] Australian Football League. Stats glossary: Every stat explained, 2017. https://www.afl.com.au/news/144837/stats-glossary-every-stat-explained. Accessed 01 Apr 2021

[26] Hopkins WG, Marshall SW, Batterham AM, Hanin J (2009). Progressive statistics for studies in sports medicine and exercise science. Med Sci Sports Exerc.

[27] Tribolet R, Sheehan WB, Novak AR, Watsford ML, Fransen J (2022). How does practice change across the season? A descriptive study of the training structures and practice activities implemented by a professional Australian football team. Int J Sport Sci Coach.

[28] Sheehan W, Tribolet R, Novak AR, Fransen J, Watsford ML (2021). An assessment of physical and spatiotemporal behaviour during different phases of match play in professional Australian football. J Sports Sci.

[29] Lane JC, van der Ploeg G, Greenham G, Norton K (2020). Characterisation of offensive and defensive game play trends in the Australian Football League (1999–2019). Int J Perform Anal Sport.

[30] Ireland D, Dawson B, Peeling P, Lester L, Heasman J, Rogalski B (2019). Do we train how we play? Investigating skill patterns in Australian football. Sci Med Footb.

[31] Browne PR, Sweeting AJ, Davids K, Robertson S (2019). Prevalence of interactions and influence of performance constraints on kick outcomes across Australian Football tiers: implications for representative practice designs. Hum Mov Sci.

[32] Mann DTY, Williams AM, Ward P, Janelle CM (2007). Perceptual-cognitive expertise in sport: a meta-analysis. J Sport Exerc Psychol.

[33] Robertson S, Back N, Bartlett JD (2016). Explaining match outcome in elite Australian Rules football using team performance indicators. J Sports Sci.

[34] Woods CT, Robertson S, Collier NF (2017). Evolution of game-play in the Australian Football League from 2001 to 2015. J Sports Sci.

[35] Young CM, Luo W, Gastin P, Tran J, Dwyer DB (2019). The relationship between match performance indicators and outcome in Australian Football. J Sci Med Sport.

[36] Fleay B, Joyce C, Banyard H, Woods CT (2018). Manipulating field dimensions during small sided games impacts the technical and physical profiles of Australian footballers. J Strength Cond Res.

[37] Hodgson C, Akenhead R, Thomas K (2014). Time-motion analysis of acceleration demands of 4v4 small-sided soccer games played on different pitch sizes. Hum Mov Sci.

[38] Gaudino P, Alberti G, Iaia FM (2014). Estimated metabolic and mechanical demands during different small-sided games in elite soccer players. Hum Mov Sci.

[39] Wing C, Hart NH, Ma’ayah F, Nosaka K (2022). Physical and technical demands of Australian Football: an analysis of maximum ball in play periods. BMC Sports Sci Med Rehabil.

[40] Thornton HR, Armstrong CR, Rigby A, Minahan CL, Johnston RD, Duthie GM. Preparing for an Australian Football League Women’s League Season. Front Sport Act Living. 2020;2 10.3389/fspor.2020.608939 PMC7785869 33426520

